# Presurgical somatostatin receptor ligand treatment does not affect tumor consistency in GH-secreting pituitary macroadenomas

**DOI:** 10.1530/EC-20-0414

**Published:** 2020-12-03

**Authors:** Marta Araujo-Castro, Héctor Pian, Ignacio Ruz-Caracuel, Alberto Acitores Cancela, Eider Pascual-Corrales, Víctor Rodríguez Berrocal

**Affiliations:** 1 Neuroendocrinology Unit, Department of Endocrinology and Nutrition, Hospital Universitario Ramón y Cajal, Instituto de Investigación Biomédica Ramón y Cajal, Madrid, Spain; 2 Endocrinology Unit, Department of Pathology, Hospital Universitario Ramón y Cajal, Madrid, Spain; 3 Neuroendocrinology Unit, Department of Neurosurgery, Hospital Universitario Ramón y Cajal, Madrid, Spain; 4 Endoscopic Skull Base Unit, Department of Neurosurgery, Hospital Universitario HM Puerta del Sur, Madrid, Spain

**Keywords:** acromegaly, fibrous tumors, tumor consistency, somatostatin receptor ligands

## Abstract

**Purpose:**

To evaluate whether presurgical treatment using long-acting somatostatin receptor ligands (SRL) may change pituitary tumor consistency and improve surgical outcome in GH-secreting pituitary macroadenomas.

**Methods:**

Retrospective study of 40 patients with GH-secreting pituitary macroadenomas operated for the first time by endoscopic transsphenoidal approach. Tumor consistency was evaluated intraoperatively and then correlated with histopathological fibrosis parameters and surgical outcomes. Surgical remission was reported based on the 2010 criteria.

**Results:**

The mean tumor size of GH-secreting macroadenomas was of 16.9 ± 8.2 mm and 25 were invasive pituitary adenomas (PAs). Presurgical treatment with long-acting SRL was performed in 17 patients (11 lanreotide, 6 octreotide). The cure rate was higher in those patients pre-treated with monthly doses ≥30 mg of octreotide or ≥90 mg of lanreotide than in those treated with lower doses or untreated (8/11 (72.7%) vs 11/29 (37.9%), *P* = 0.049). However, although the proportion of soft tumors increased as higher doses of SRL were considered in the pre-treated group, no statistical significance was reached, even when the highest approved monthly doses were used (6/6 (100%) vs 23/34 (67.7%), *P* = 0.102). Moreover, we found that the remission rate was similar between fibrous and soft tumors (*P* = 0.873) and also of surgical complications (*P* = 0.859), despite of the higher prevalence of Knosp >2 (*P* = 0.035) and very large PA (*P* = 0.025) in fibrous tumors than in soft tumors.

**Conclusions:**

Although presurgical treatment with high doses of SRL was associated with a 2.2-fold greater chance of surgical remission, this benefit was not related with changes in tumor consistency induced by the presurgical treatment.

## Introduction

Acromegaly is a rare disease characterized by the overproduction of growth hormone (GH), which is commonly secreted by a pituitary adenoma (PA). Because of cardiovascular, respiratory, and metabolic comorbidities, patients with active acromegaly are associated with higher mortality (1). Transsphenoidal surgery is generally considered the first-line treatment of choice in acro-megaly (1). However, although surgical remission is achieved in up to 100% of microadenomas in reference centers (2), there is a high risk of not achieving cure in macroadenomas, which account for up to 77% (3), and in invasive PAs. In these group of patients, remission rate is of 60–70% and 10–30%, respectively, in hands of experienced neurosurgeons (2, 4).

There are several studies that have been focused on predictive factors of surgical remission in acromegaly (2, 5, 6). Classical factors are tumor size (7), GH levels (2, 5), Knosp grade (2, 6), and age (2). Moreover, tumor consistency was reported to be an important predictive factor since hard consistency could impede transsphenoidal resection, especially in macroadenomas (8). Preoperative somatostatin receptor ligands (SRLs) treatment is proven to reduce GH and IGF1 levels (9) and induce tumor shrinkage (10), improving surgical outcomes in acromegaly (11, 12, 13). However, the impact of SRLs in the GH-secreting pituitary tumor consistency has yet to be elucidated, and controversial results have been found (14, 15, 16, 17).

In this way, we aimed to determine if tumor consistency could be affected by presurgical medical treatment with long-acting SRLs in GH-pituitary macroadenomas, and if tumor consistency is associated with surgical outcomes, including surgical remission and complications.

## Methods

### Patients

A retrospective study of patients with GH-secreting PAs operated by endonasal endoscopic transsphenoidal approach and for the first time at the Hospital Ramón y Cajal and Hospital HM Puerta del Sur was carried out. Fifty-six patients with acromegaly, operated between 2008 and 2019, were identified. Those patients who met inclusion criteria and not presented exclusion criteria were enrolled in the study. Inclusion criteria were: (i) Confirmed acromegaly diagnosis (GH levels >1 ng/mL after oral glucose tolerance test (OGTT)) and fasting plasma IGF1 levels above reference ranges for age and sex (1), (ii) operated by the senior author and (iii) with a tumor size ≥10 mm. Exclusion criteria were: (i) Previous pituitary surgery , treatment with dopamine agonists and/or radiotherapy (*n* = 3) and (ii) not available information of clinical, hormonal, radiological or histological tumor characteristics, (iii) operated by other neurosurgeons (*n* = 4) and (iv) tumor size <10 mm (*n* = 8). Patients with microadenomas were excluded in our study since one of our purposes was to evaluate the possible relation between SRL pre-treatment and surgical remission, and it is known that surgical cure is achieved in nearly 100% of GH-secreting pituitary microadenomas (2). A total of 40 patients were included. Two groups were established based on presurgical treatment with SRL: pre-treatment group, *n* = 17 (patients pre-treated with long -acting SRL for equal or longer than 3 months) and untreated group, *n* = 23 (patients never treated with SRL before surgery or in whom the duration of SRL therapy was less than 3 months). There was only one patient in the untreated group that had been received one doses of 30 mg of octreotide.

The pituitary tumors register was approved by the local ethical committees of both hospitals (Ethical committe of the Hospital Universitario Ramón y Cajal and ethical committee of Hospital HM Puerta del Sur).

### Assays and remission definition

All anterior pituitary hormones including GH and IGF1 were measured pre- and post-operatively following our protocol (18). Presurgical measurements were performed at time of diagnosis, before any medical or surgical treatment. GH and IGF1 were measured by chemiluminescence assays. They were measured with IMMULITE 2000 before May 2013; with Isys (IDS Vitro) between May 2013 and October 2018 and by Liaison XL (Diasorin) after then. The intraassay coefficient of variation (CV) was <10% with all methods. The assays were calibrated according to the WHO international standard for GH and IGF1 with code 98/574 and 02/254, respectively.

We have evaluated surgical remission at least 3 months after surgery (and at least 4 months in those pre-treated with SRL (19)), using the 2010 criteria of the AACE guidelines (random GH <1 ng/mL or GH nadir <0.4 ng/mL, on OGTT, along with a normal age- and sex-matched IGF1) (20).

### Radiological assessment

Imaging studies were performed with MRI with 1.5T, GE 450w. Sagittal and coronal T1, T2-weighted and dynamic sequences, with gadolinium contrast was performed preoperatively and before any medical or surgical treatment, and 3–6 months postoperatively. Depending on tumor size, PA was classified in macroadenoma (≥10 mm), very large PA (≥30 mm) and giant PA (≥40 mm). Invasive PAs were defined as tumors with Grade 3 or 4 of the classification of Knosp (21).

### Surgical procedure and histological analysis

A conventional transsphenoidal endoscopic endonasal approach was used in all surgeries. The approach included a binarial four-hand technique with wide anterior sphenoidotomy and partial posterior septectomy. Extended approaches were performed in case of cavernous sinus invasion. Surgically removed specimens were immediately fixed in 10% buffered formalin and subsequently embedded in paraffin. Standard H & E-stained sections were used for diagnosis. Specimens were classified based on the 2017 WHO Classification (22) and the grade of fibrosis based on the percentage of collagen (1: <5%, 2: 5–15% and 3: > 15%) in relation to the whole area of tissue stained with hematoxylin-eosin (23). Tumors were classified in two groups: soft tumors if they were easily suckable and hard/fibrous tumors when they need to be fragmented for removing using ring curettes. In addition, within hard tumors we have analyzed a subgroup of very hard tumors, in which for their removal we have had to fragment using sharp dissections or ultrasonic surgical aspirators.

### Statistical analysis

The statistical analysis was performed with STATA.15. In the descriptive analysis, categorical variables were expressed as absolute values and proportions of the variable; quantitative variables were expressed as mean ± s.d. or as medians ± interquartile ranges (IQR) depending whether the normality assumption was fulfilled. The normality assumption was studied with Shapiro-Wilk test and the variance homogeneity assumption with the Levene test. For the comparison of differences in continuous parameters, student’s *t*-test was performed, and for the comparison of categorical variables between independent samples, the chi-squared-test. Multivariant logistic regression model was performed for the identification of variables associated with surgical remission. Ordinal correlations were calculated with Kendall correlation test based on tau-b (corrected by ties). In all cases, a two-tailed *P* value <0.05 was considered as statistically significant.

## Results

### Baseline characteristics

The clinical characteristics of all 40 patients with acromegaly enrolled in this study are shown in Table 1. The mean tumor size was of 16.9 ± 8.2 mm, 25 of them were invasive macroadenomas. Seventeen patients were treated with SRLs in the preoperative stage (Octreotide LAR i.m., 6 cases; Lanreotide Autogel s.c., 11 cases) for at least 3 months preoperatively (median, 6.0 months; range, 3–84 months). Median monthly doses were of 90 mg (range 60–120) with lanreotide and 20 mg (range 20–30) for octreotide, 11 of the 17 patients (64.7%) were treated with monthly doses of octreotide ≥30 mg or lanreotide ≥90 mg and 6 patients with ≥30 mg of octreotide or ≥120 mg of lanreotide. Presurgical variables were comparable between patients pre-treated with SRLs and untreated, except in the prevalence of headache and visual involvement that was higher in the untreated group, probably in relation to a slightly greater nonsignificant proportion of very large PA (≥30 mm) in this group.

**Table 1:** Characteristics of patients in the pre-treated and untreated group.

Variables	Untreated (*n* = 23)	Pre-treated (*n* = 17)	*P* value
Age (years)	48.0 ± 13.0	51.2 ± 13.8	0.455
Female sex	15/23	12/17	0.720
Diabetes	2/23	4/17	0.194
Hypertension	6/23	6/17	0.530
Heart disease	1/23	1/17	0.826
Obesity	3/23	2/17	0.904
SAS	5/23	3/17	0.749
Pituitary apoplexy	1/23	0/17	0.384
Visual involvement	5/23	0/17	**0.040**
Headache	9/23	1/17	**0.030**
Hypopituitarism	6/23	3/17	0.527
Presurgical GH	13.0 ± 17.5	10.9 ± 8.7	0.641
Presurgical IGF1	693.0 ± 316.8	653.1 ± 260.1	0.674
Very large PA	4/23	0/17	0.070
Tumor size at diagnosis (mm)	18.3 ± 8.5	15.1 ± 7.7	0.223
Knosp grade 3–4	11/23	4/17	0.117

Presurgical GH and IGF1 are expressed in ng/mL and refers to the values previous to SRL treatment. Bold values indicate statistical significance.

SAS, sleep apnoea syndrome.

### Tumor consistency according to presurgical treatment

**Figure 1:**
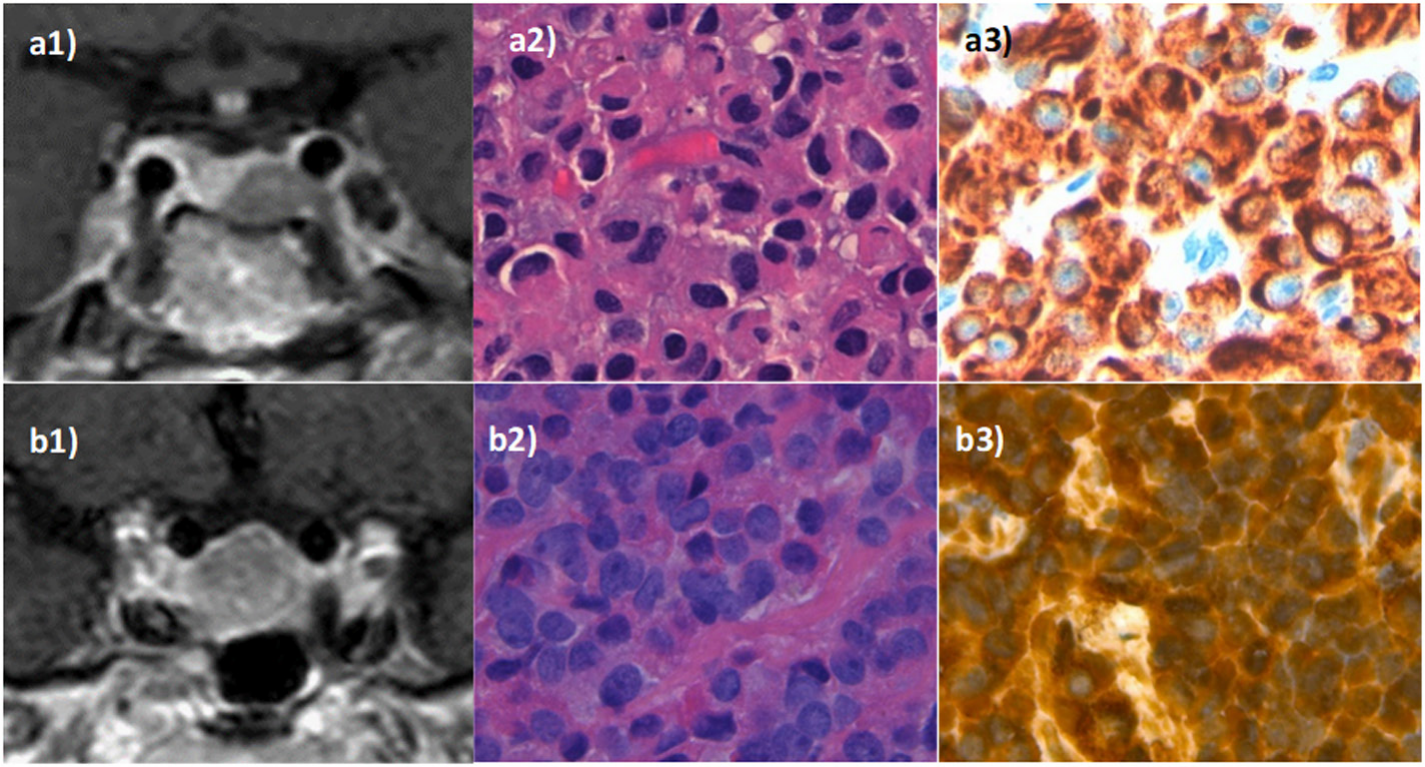
Radiological and histological images of soft and hard tumors. Image a1 represents a soft Knosp grade 0 GH macroadenoma in a patient pre-treated with somatostatin receptor ligands (SRL) for 84 months, surgical remission was achieved (coronal T2 sequence). This pituitary adenoma presented high cellularity separated by fine connective tracts (a2), and strong and diffuse positivity for GH (a3) in histological exams. Image b1 (T1 coronal with gadolinium administration) shows a very hard Knosp grade 2 GH macroadenoma tumor in a patient not pre-treated with SRL. He was cured after surgery. This tumor presented high cellularity separated by fibrous septa (b2) and diffuse and strong positivity for GH (b3) in histological exams.

Most GH-secreting PAs were of soft consistency, 29 of the 40 PAs (72.5%), and the remaining 11 PAs (27.5%) were of fibrous consistency (hard consistency in eight PAs and very hard in three) (Fig. 1). Globally, no differences were found in the proportion of soft tumors between pre-treated and untreated patients (14/17 (82.4%) vs 15/23 (65.2%), *P* = 0.230). However, when higher doses of SRLs were considered (patients pre-treated with monthly doses of octreotide ≥30 mg or lanreotide ≥90 mg compared with treated with lower doses or untreated), there was a no significant higher number of soft tumor in the pre-treated group (10/11 (90.9%) vs 19/29 (65.5%), *P* = 0.108). Moreover, if the highest monthly doses were used (≥ 30 mg of octreotide or ≥120 of lanreotide), 6 of the 6 of pre-treated patients were of soft consistency vs 23 of 34 in those treated with lower doses or untreated, but no statistical significance was reached (*P* = 0.102). Nonetheless, despite a tendency to a negative correlation between SRL doses and the degree of tumor hardness, no statistical significance was achieved (Kendalls‘s tau-b = -0.31, *P* = 0.166).

### Other presurgical variables associated with tumor consistency

Fibrous consistency was associated with cavernous sinuses invasion, as Knosp 3–4 PAs were significantly more prevalent in fibrous PAs than soft PAs (7/11 (63.6%) vs 8/29 (27.6%), *P* = 0.035). Besides the proportion of very large PAs (3/11 (27.3%) vs 1/29 (3.5%), *P* = 0.025) and mean tumor size was significantly higher (21.9 ± 2.3 vs 15.0 ± 1.4 mm, *P* = 0.016) in fibrous tumors than soft tumors. No other differences were found between the two groups (Table 2).

**Table 2:** Presurgical variables associated with tumor consistency.

Variable	Soft tumors (*n* = 29)	Fibrous tumors (*n* = 11)	*P* value	OR (95% CI)
Age (years)	51.1 ± 13.2	44.6 ± 12.7	0.172	1.0 (0.9–1.0)
Female sex	20/29	7/11	0.748	0.8 (0.2–3.4)
Diabetes	1/29	5/11	0.519	0.5 (0.0–4.6)
Hypertension	10/29	2/11	0.315	0.4 (0.1–2.3)
Heart disease	2/29	0/11	0.372	NC
Obesity	2/29	2/11	0.082	5.1 (0.7–35.8)
SAS	6/29	2/11	0.859	0.9 (0.1–5.0)
Pituitary apoplexy	0/29	1/11	0.100	NC
Visual involvement	2/29	3/11	0.082	**0.2 (0.0–1.4)**
Headache	3/29	6/11	**0.003**	**0.1 (0.0–0.5)**
Hypopituitarism	15/29	4/11	0.385	0.5 (0.1–2.2)
Presurgical GH	14.2 ± 15.7	6.8 ± 7.7	0.150	0.9 (0.8–1.0)
Presurgical IGF1	695.5 ± 301.2	624.5 ± 269.2	0.498	1.0 (1.0–1.0)
Very large PA	1/29	3/11	**0.025**	0.1 (0.0–1.0)
Tumor size (mm)	15.0 ± 7.7	21.9 ± 7.8	**0.016**	1.1 (1.0–1.2)
Knosp grade 3–4	8/29	7/11	**0.035**	0.2 (0.0–0.9)

OR has been calculated as a measurement of association with soft consistency. Bold values indicate statistical significance.

### Surgical outcomes according to presurgical treatment and tumor consistency

After median follow-up of 59.2 (range 8.8 to 147.3) months, surgical remission was achieved in 19 of the 40 patients and in 2 of the 15 invasive PAs. The cure rate was higher in those pre-treated with monthly doses of SRL ≥30 mg of octreotide or ≥90 mg of lanreotide than in those treated with lower doses or untreated (8/11 (72.7%) vs 11/29 (37.9%), *P* = 0.049), but no benefit of lower doses was observed. No differences in surgical and endocrine complications were observed between these two groups (1/11 (9.1%) vs 7/29 (24.1%), *P* = 0.288) nor in other clinical, radiological and histological characteristics (Table 3). The only variable independently associated with surgical remission in the multivariant logistic regression model was Knosp grade <3 (OR = 9.4, *P* = 0.020).

**Table 3:** Differential features of patients treated with high doses of SRL and those untreated or treated with low doses.

Variable	High doses (*n* = 11)	Low doses and untreated (*n* = 29)	*P* value
Age (years)	51.4 ± 13.1	48.6 ± 13.5	0.556
Female sex	7/11	20/29	0.748
Visual involvement	0/11	5/29	0.141
Hypopituitarism	5/11	14/29	0.873
Presurgical GH	9.4 ± 8.2	13.2 ± 16.0	0.466
Presurgical IGF1	608 ± 274.6	701.8 ± 297.7	0.370
Tumor size (mm)	14.2 ± 7.8	18.0 ± 8.3	0.199
Very large PA	0/11	4/29	0.194
Hard consistency	1/11	10/29	0.108
Fibrosis grade 2–3^a^	2/8	5/11	0.361

^a^Information about the grade of fibrosis was available only in 19 patients.

Based on tumor consistency, no differences were found in surgical remission between fibrous and soft tumors (5/11 (45.5%) vs 14/29 (48.3%), *P* = 0.873). The rate of surgical complications was no different between fibrous and soft tumors (2/11 (18.2%) vs 6/29 (20.7%), *P* = 0.859). No differences were found either when the subgroup of very hard tumors vs hard and soft tumors were compared, in surgical remission (*P* = 0.489) nor in rate of complications (*P* = 0.368).

### Histopathological features associated with tumor consistency

Positive immunostaining for GH was demonstrated 25 patients (14 for GH and PRL, 1 for TSH and GH and 10 only for GH); in 13 patients the GH IHQ was negative or IHQ was not performed; in one patients IHQ was only positive for prolactin and in the other one only for TSH. Ki67 >3% was observed in five PAs.

Information of grade of fibrosis was available in 19 PAs, seven of them presented grade 2 o 3 of fibrosis. The proportion of tumors with a grade 2 or 3 of fibrosis tended to be higher in hard tumors than in soft, but not statistical significance was reached (2/3 (66.7%) vs 5/16 (32.3%), *P* = 0.243). Based on the WHO 2017 classification (available in 17 cases), the most common subtype of GH-secreting PA was the densely granulated somatotroph adenoma (8/17), followed by sparsely granulated somatotroph adenoma (5/17) and mixed somatotroph-lactotroph adenoma (4/17) (Fig. 1).

## Discussion

We found that long-term surgical remission occurs significantly more frequently in GH-secreting pituitary macroadenomas pre-treated with SRLs at monthly doses ≥30 mg of octreotide or ≥90 mg of lanreotide than in treated with lower doses or untreated. However, no differences in the proportion of soft tumors were observed between pre-treated patients and untreated. Moreover, tumor consistency was not associated with surgical remission or perioperative morbidity.

The percentage of hard tumors GH-secreting macroadenomas in our series was of 27.5% (11 out of 40), and of very hard of 7.5% (3 out of 40). This prevalence is slightly higher that the generally considered in most series, to be between 5 and 13.5% (24, 25, 26), or even some studies reported prevalence of 0% in this type of functioning-PAs (24, 27). In general, functional tumors were of softer consistency than non-functional PAs (26). These differences in prevalence are probably related to the wide range of definitions used, since most definitions are based on intraoperative findings. Therefore, classification depends on neurosurgeon criteria and varies from two grade consistence classification (24, 25) to even five-grade classification (26).

We did not find differences in the proportion of soft tumors between pre-treated and untreated patients, but as the doses of SRLs increased the proportion of soft tumors tended to increase too, from 14/17 (82.4%) with conventional doses, to 10/11 (90.9%) with middle doses and 6/6 (100%) with the highest approved SRLs doses. Other authors did not find significant changes in the tumor consistency with the pre-treatment with SRLs either (14). However, an increased proportion of soft tumors in patients pre-treated with SRLs has been reported in several previous studies (16, 28). Our hypothesis is that the ability of SRL to reduce GH and IGF1 levels, shrink the tumor and soften tumor consistency depends on the doses of SRLs, so high doses of SRLs would be necessary for this purpose. This theory is supported by the increased proportion of soft tumors observed as doses of SRLs increased, although no statistical significance was reached, probably due to the limited sample size.

We did not demonstrate that tumor consistency affects surgical outcomes, as remission and surgical complications were similar between fibrous and soft PAs, despite of the higher proportion of invasive and very large PAs in the subgroup of fibrous tumors than in soft tumors. Our results are supported by previous studies, finding similar surgical results in soft an fibrous tumors (13) or even some authors (29) suggesting that the firm capsule may facilitate the removal of a fibrous suprasellar adenoma. Moreover, other authors have previously described that PAs invading the cavernous sinuses had a higher consistency grade (26), but also a greater risk of surgical morbidity and lower chance of surgical remission in this series. In this same line, most literature agrees that treating fibrous tumors are associated with certain technical problems, including the need for two-stage transsphenoidal surgery (8, 30), and thus a subsequently lower chance of total surgical resection and higher risk of complications, similarly as the observed in meningiomas (31). Our results support that tumor consistence is not a predictive factor of surgical outcomes by itself, but its association with higher invasiveness and tumor size could justify the higher morbidity and lower chance of tumoral resection depending on tumor consistency. In this way, neurosurgeon experience plays an important role in surgical outcomes, especially in these more complicated cases.

A clear benefit of presurgical SRLs was observed when doses ≥30 mg of octreotide or ≥90 mg of lanreotide were used, since surgical remission was 2.2-fold times greater in pre-treated patients than treated with lower doses or untreated. Several studies concluded that pre-treatment with SRLs improves surgical remission rate (32, 33) and it was probably related with a reduction of presurgical IGF1 and GH levels (9) and tumor size (10). Some studies showed that only macroadenoma and invasive macro- or giant PAs benefited from the SRLs pre-treatment (32), whereas others reported advantage in all patients with acromegaly (34). However, other studies did not demonstrate significant beneficial effects of SRL pre-treatment on surgical outcome (35). Nevertheless, differences in these studies could be justified by different study populations, different surgical remission definitions and not the same formulations and doses of SRLs used in each study, among other issues. High SRL doses could increase its affinity for somatostatin receptor 5 or induce an up regulation of somatostatin receptor 2 or alter its degradation (36). Thus, higher doses of SRL may improve surgical outcome (36). This is supported by previous studies that have shown that the serum concentrations of SRL correlate with their long-term efficacy against GH hypersecretion (37). However, further studies comparing different doses of SRLs pre-treatment are needed to clarify its effect on surgical outcomes.

As limitations of our study, it is retrospectively designed from 2008 to 2012, and the number of patients was relatively small, so the possibility of a type 2 error should be considered and the potency of the study was lower than 80% to demonstrate differences in tumor consistency between pre-treated and untreated patients, so a prospective study and with a large number of patients should be performed to confirm our results.

## Conclusion

Although presurgical treatment with high doses of SRLs was associated with a 2.2-fold greater chance of surgical remission in GH-pituitary macroadenomas, this benefit was not related with changes in tumor consistency induced by the presurgical treatment. Tumor consistency was not associated with surgical outcomes, including surgical remission and complications.

## Declaration of interest

The authors declare that there is no conflict of interest that could be perceived as prejudicing the impartiality of the research reported.

## Funding

IRYCIS: Convocatoria intramural de ayudas a proyectos de investigación de investigadores noveles, investigadores clínicos asociados y/o grupos emergentes del Hospital Universitario Ramón y Cajal.

## Ethical approval

All procedures performed in the participants of the study were in accordance with the ethical standards of the institutional research committee and with the 1964 Helsinki declaration and its later amendments or comparable ethical standards.

## Informed consent

Informed consent was obtained from all individual participants included in the study.
